# Notoginsenoside R1 (NG-R1) Promoted Lymphatic Drainage Function to Ameliorating Rheumatoid Arthritis in TNF-Tg Mice by Suppressing NF-κB Signaling Pathway

**DOI:** 10.3389/fphar.2021.730579

**Published:** 2022-02-24

**Authors:** Danli Jiao, Yang Liu, Tong Hou, Hao Xu, Xiaoyun Wang, Qi Shi, Yongjun Wang, Qiujuan Xing, Qianqian Liang

**Affiliations:** ^1^ Longhua Hospital, Shanghai University of Traditional Chinese Medicine, Shanghai, China; ^2^ Shanghai Changning Tianshan Traditional Chinese Medicine Hospital, Shanghai, China; ^3^ Spine Institute, Shanghai University of Traditional Chinese Medicine, Shanghai, China; ^4^ Key Laboratory of Theory and Therapy of Muscles and Bones, Ministry of Education (Shanghai University of Traditional Chinese Medicine), Shanghai, China; ^5^ Shanghai Research Institute of Acupuncture and Meridian, Shanghai University of Traditional Chinese Medicine, Shanghai, China

**Keywords:** notoginsenoside R1, rheumatoid arthritis, lymphatic drainage function, NF-κB signaling pathway, inflammation, TNF-Tg mice

## Abstract

Rheumatoid arthritis (RA) is a chronic autoimmune disease that is primarily characterized by synovial inflammation. Our previous studies demonstrated that the lymphatic system is critical for the development and maintenance of RA disease, and sufficient lymph drainage helps to improve joint inflammation. In this study, we found that NG-R1, the main active component in the traditional Chinese medicinal herb Sanchi, activating lymphatic function can attenuate synovial inflammation. According to histopathological staining of ankle sections, NG-R1 significantly decreased the area of inflammation and reduced bone destruction of ankle joints in TNF-Tg mice. Near infrared-indocyanine green (NIR-ICG) lymphatic imaging system has shown that NG-R1 significantly improved the lymphatic drainage function. However, the molecular mechanism of its activity is not properly understood. Our in-depth study demonstrates that NG-R1 reduced the inflammatory cytokine production of lymphatic endothelial cells (LECs) stimulated by TNF-α, and the mechanism ameliorated the phosphorylation of IKKα/β and p65, and the translocation of p65 into the nucleus. In summary, this study proved that NG-R1 promoted lymphatic drainage function to ameliorating rheumatoid arthritis in TNF-Tg mice by suppressing NF-κB signaling pathway.

## Introduction

Rheumatoid arthritis is a chronic systemic disease with autoimmune pathogenesis and systemic involvement, which is associated with the overexpression profile of many proinflammatory cytokines (Petrovska et al., 2021, 102797; Hosseinikhah et al., 2021). This autoimmune disorder affects nearly 1% of the total adult population and remains a cause of high rates of morbidity and mortality in humans (Zhao and Zhang, 2018, 123). NF-κB activation contributes to the inflammatory pathogenesis of RA, resulting in synovium hyperplasia and cartilage and bone erosion (Wang et al., 2016; Zhang et al., 2018; Yang et al., 2019; Acimovic et al., 2018). Thus, the potential treatment of RA by blocking NF-κB activation interactions can be highlighted. Current RA therapies mainly target immune inflammation and the efficacy is subject to ceiling effects (Pratt et al., 2021, e337–e346).

The lymphatic system plays a key role in the pathogenesis and treatment of inflammatory and invasive arthritis, and it has been proved that improving lymphatic drainage function effectively reduced RA joint inflammation (Bouta et al., 2018, 94–106; Hou et al., 2020, 526–534; Liu et al., 2021, 541–553). The lymphatic system is involved in the pathologic development of rheumatoid arthritis by redistributing cytokines, chemical factors, and other inflammation-related factors around the inflamed joint into the lymphatic vessels, thereby reducing the inflammatory factors in the joint cavity aggravated by accumulated joint lesions (Liang et al., 2015a, 648–55). Specifically, long-term chronic inflammation will stimulate lymphatic endothelial cells (LECs) to release proinflammatory cytokines, which will accelerate the apoptosis of lymphatic smooth muscle cells (LSMCs) and inhibit their proliferation (Telinius and Hjortdal, 2019, 1777–1784, Liang et al., 2021, 58). This persistent inflammation leads to structural and functional of lymphatics abnormalities, lymphatic drainage dysfunction, and further aggravates the inflammation of the joints (Deng et al., 2021, 2174; Liang et al., 2020, e12876). Most importantly, targeting lymphatics represents an innovative strategy for therapeutic intervention in RA.

*Panax notoginsen* (Burk) F. H. Chen (Sanqi in Chinese) is a traditional Chinese medical drug which has the effect of promoting blood circulation and removing blood stasis (Wang et al., 2016, 234–58). Notoginsenoside R1 (NG-R1) is one of the major bioactive ingredients of Panax notoginsen. Modern pharmacological studies have indicated that NG-R1 possesses anti-tumor, anti-aging, antioxidative, and anti-inflammation effects (Sun et al., 2013, 1758–70, Yu et al., 2016, 21730; Liu et al., 2020, 551–565). However, it is not clear whether NG-R1 can alleviate arthritis inflammation in RA and whether it has beneficial effects on lymphatic function. We in a preliminary experiment found that NG-R1 had anti-inflammatory and anti-rheumatic effects. Therefore, we conducted *in vivo* and *in vitro* experiments to verify that NG-R1 reduces articular inflammatory injury and alleviates RA progression by promoting lymphatic drainage function.

## Materials and Methods

### Animals

Three-month-old female TNF-Tg (TNF-Tg line 3,647) mice were provided by the Shanghai Southern Model Animal Laboratory Center (Shanghai, China). The TNF-Tg mice were bred as heterozygotes on a C57BL/6 background, and their wild-type (WT) littermates were used as normal controls. Mice were housed in a temperature (25°C) and humidity (45–55%)-controlled environment and provided libitum access to maintenance diet and water. All animal procedures were performed according to the Guiding Principles for the Care and Use of Laboratory Animals approved by the Animal Regulations of the National Science and Technology Committee of China under the approval of the Longhua Hospital Animal Ethics Committee (Shanghai, China).

### Drug Intervention

TNF-Tg mice were randomly divided into two groups: TNF-Tg group and NG-R1 group (10 mice/group). A batch of littermates of wild type (WT) mice were used as the WT group (10 mice/group). The TNF-Tg group and WT group were treated with the same volume of 0.5% CMC-Na (0.5 g CMC-Na + 99.5 ml aqueous solution + 500 µl tween). NG-R1 group mice were treated with 20 mg/kg NG-R1 (2 mg NG-R1 + 1 ml 0.5% CMC-Na) by daily intraperitoneal injection and continuously for 8 weeks.

### Arthritis Symptoms Assessment

We conducted the measurements of arthritis index, ankle circumference, and weight. Arthritis disease malformation grade assessment: arthritis grade 0, normal (no swelling or malformation); grade 1, mild, but marked redness of the ankle joint or wrist swelling, or significant redness and swelling or malformation limited to individual figures, regardless of the number of affected numbers; grade 2, moderate redness and swelling or malformation of the ankle or wrist; grade 3, severe redness and swelling or malformation of the entire paw, including number; grade 4, the limbs are most inflamed and multiple joints. Body weight: an electronic body weight scale will measure weight once a week. Ankle circumference: the degree of ankle joint swelling was measured with a vernier caliper. The counts and grades recorded by two independent blinded examiners once a week.

### Near-Infrared Indocyanine Green Lymphatic Imaging

Mice were anesthetised with isoflurane and treated with local depilation, then ICG (0.1 μg/ml, dissolved in distilled water and stored at 4°C in the dark) was injected into footpads. After the ICG signal was stable, the fluorescence signal was observed for 600 s continuously, and the pulse number was obtained according to the fluctuation times of the signal intensity of lymphatic vessels. The ImageJ software (National Institutes of Health, Bethesda, Maryland) was used to determine the region of interest (ROI) during the lymph flow in the leg of mice, and the fluorescence signal intensity within the ROI was recorded at 1 and 24 h. Lymphatic clearance was calculated according to the following formula: clearance = ((ROI1h−ROI1hbg)-(ROI24h− ROI24hbg))/(ROI1 h − ROI1 hbg)*100% (bg = background).

### Histologic and Histomorphometric Analyses

After ending treatments, the mice were sacrificed, and the ankle joints were excised to prepare paraffin sections. Four-micrometer consecutive tissue sections were cut from each arrayed paraffin block and prepared on pathological slides. The slices were stained by alcian blue/orange G (ABOG) or tartrate-resistant acid phosphatase (TRAP) for histologic analysis. The inflamation area, cartilage area, osteoclast number at ankle joints, and astragalus bone area of the ankle joint of mice in 3 groups were statistically analyzed. Slides were scanned using the Olympus VS120-S5-E whole-slide imaging system (Olympus, Japan). All images were analyzed using the Olympus OlyVIA software (Olympus, Japan).

### Immunofluorescence and Immunohistochemistry

The number of lymphatic vessels was counted by immunofluorescence staining. The tissue sections were added with trypsin and repaired in 37°C incubators for 15 min, blocked by 4% Bovine Serum Albumin (BSA) for another 1 h. Primary FITCα-SMA antibody (Sigma, F3777) and hamster monoclonal antimouse podoplanin antibody (Abcam, cat. #ab11936) incubate overnight at 4°C. The second Goat anti-hamster podoplanin (1:400, diluted by 0.4%BSA/PBS reagent) was incubated for another 1 h and immunofluorescence staining slides were scanned using an Olympus VS-120 whole-slide imaging system.

Whole-mount staining of the back skin of the foot was stained to analyze the percentage of smooth muscle cell (SMC) coverage. The skin of the back of the foot was obtained when mice were sacrificed. We removed fat and other connective tissue, and then soaked the kin in 10% formalin at 4°C overnight and then permeated with 0.5% Triton X. After blocking with 4% Bovine Serum Albumin (BSA) for another 1 h, the skin tissues were incubated with 1: 1,000 dilution of Podoplanin antibody (Abcam, cat. #ab11936) and 1:400 dilution of fluorescein isothiocyanate labeled anti-SMA antibody at 4°C overnight. The images were recorded by Olympus VS-120 software. The whole operation should avoid light. The calculation formula is as follows: SMC coverage percentage = (SMC coverage area/whole lymphatic vessel area) × 100%.

### Micro-Computed Tomography

The tight ankle joints were obtained when mice were sacrificed and then fixed in 10% formalin for 48 h, washed in phosphate-buffered saline (PBS) for 2 h, and then soaked in 75% ethanol, scanned by micro-CT system (Scanco VIVA CT80, SCANCO Medical AG, Switzerland). The X-ray tube voltage was 55 kV and tube current 72 μA, with a voxel size of 10 µm. The cross-section images were realigned in 3D using the SCANCO proprietary software to observe the morphology of astragalus.

### Serum Biochemical Parameters Determination

Peripheral blood samples were collected from mice when they were sacrificed. Blood samples (1 ml each) were collected from mouse eyeballs and then centrifuged to extract the upper serum. The serum level of Inflammatory cytokines TNF-α (NeoBioScience, China. cat.# EMC102a) and IL-6 (NeoBioScience, China. cat. #EMC004) were detected in strict accordance with the instructions of the kit.

### Cell Culture

Mouse Primary Lymphatic Endothelial Cells/C57-6092 (Catalog Number: C57-6092) were cultured in α-MEM (Hyclone, Logan, UT, United States) supplemented with 100 IU/ml of penicillin/streptomycin (Sigma-Aldrich, United States) and 10% heat-inactivated fetal bovine serum (Gibco, United States) and maintained at 37°C in a humidified atmosphere with 5% CO_2_.

### Cytotoxicity Assay

Cytotoxicity assay was measured by Cell Counting Kit-8 (Dojindo. Japan. cat. #CK04) assay according to the manufacturer’s instructions. LECs were adjusted at a density of 1 × 10^4^ cells/ml seeded in a 96-well plate, then exposed at various concentrations of NG-R1 for 24 h. A total of 10 µl CCK-8 solution was added into each well for another 1 h at 37°C. The resulting optical density was detected at 450 nm by a microplate reader (Biotek).

### Co-Culture of LECs and LSMCs

Co-culture was performed with Transwell (0.4 μm pore size, Corning). LECs were seeded on chambers in 6-well plates for 24 h, and then pretreated with NG-R1 (5, 10, and 50 µM) for 2 h, stimulated and then pretreated with NG-R1 (5, 10, 50 µM) for 2 h and stimulated with TNF-α, culture chambers with LECs were then transferred into another 6-well plate, which had already been coated with LSMCs for 2 days. After 24 h of co-culture, LSMCs were harvested for analysis of the expression level of functional muscle genes h1-calponin, smα22, SM myosin heavy chain, and smα2 mRNA.

### Real-Time Polymerase Chain Reaction

Total RNA was extracted using Trizol (Thermo.United States. cat. #15596018) reagent according to the manufacturer’s instructions and the cDNA obtained by using the PrimeScript TM RT kit (Taraka. Japan. cat. #RR037A) according to the manufacturer’s protocol. Real-time PCR analysis was performed using Hieff TM qPCR SYBR Green Master Mix (Taraka. Japan. cat. #1 1201ES08) reagents on q-PCR CFX96 machine (Bio-rad). The primers for qRT-PCR were shown in Table 1.

**TABLE 1:** Sequence of primers used in the real time polymerase chain reaction.

Genes	Sequence of primers	Gen bank accession number	Annealing (°C)	Product size (bp)
h1-caIponin	F: 5′ TGGCCCAGA AATACGACCAC3′	NM 031747.1	60	144
	R: 5′ CCG​GCT​GGA​GCT​TGT​TGA​TA3′			
SM α22-actin	F: 5′ TCT​CCT​TCC​AGC​CCA​CAA​AC3′	NM_031549.2	60	82
	R: 5′ TTC​ACG​GCT​CAT​GCC​ATA​GG3′			
Smooth muscle myoxln, heavy chain 11	F: 5′ TCC​GGT​GTT​CTC​CTG​CTA​GT 3′	NM_001170600.1	60	134
	R: 5′ GGG​CCA​TTG​GGC​TGT​TTA​TG 3′			
SM α2-action	F: 5′ TAT​TCT​GTC​TGG​ATC​GGC​GG 3′	NM_031004.2	60	196
	R: 5′ ACA​TTC​ACA​GTT​GTG​TGC​TAG​AG 3′			
TNF- α	F: 5′ AGTGACAAGCCTGTA GCCC 3′	NM_013693.3	57	252
	R: 5′ GAG​GTT​GAC​TTT​CTC​CTG​GTA​T 3′			
IL-1β	F: 5′ CTGCTACATCA GCACCTCAC 3′	NM_008361.4	55	124
	R: 5′ AGAAACAGTCCA GGCCATAC 3′			
IL-6	F: 5′ TGT​ATG​AAC​AAC​GAT​GAT​GCA​CTT 3′	NM_001314054.1	60	197
	R: 5′ ACTCTGGCTTTG TCTTTCTTGTTATCT3′			
iNOS	F: 5′ AACGGA GAAC GTTGGATTTG 3′	NM_010927.3	54	151
	R: 5′CAG​CAC​AAG​GGG​TTT​TCT​TC3′			
β-Actin	F: 5′ TTGCTGACA GGATGCA GAAGGAGA 3′	NM_031144.3	60	159
	R: 5′ ACT​CCT​GCT​TGC​TGA​TCC​ACA​TCT 3′			

### Western Blot

The protein was collected, and 30 µg of protein from each sample was separated by 15% SDSPAGE and transferred to polyvinylidene flfluoride membrane and then separated by sodium dodecyl sulfate polyacrylamide gel electrophoresis (SDSPAGE) and transferred to a Polyvinylidene flfluoride (PVDF) membrane. After blocking with 5% bovine serum albumin in TBST at room temperature for 2 h, the membranes were incubated with the Phospho-IKKα (Ser176)/IKKβ (Ser177) Antibody (cat. # 2688), Phospho-NF-κB p65 (Ser536) (93H1) Rabbit mAb (cat. #3033) overnight at 4°C. After washing with TBST for three times, the membranes were incubated with the corresponding horseradish peroxidase-labeled secondary antibodies (cat. #7074). Proteins were scanned using the ECL detection system. All the antibodies were purchased from Cell SignalingTechnology (Beverly, NJ, United States).

### Statistical Analysis

Statistical analysis was performed by Graphpad Prism (Version 8.0). Data are presented as means ± standard error of mean (SEM). One-way ANOVA followed by Dunnett’s t-test was used to determine differences between groups. As to 3 groups with different time points, we applied two-way ANOVA (or nonparametric) comparison. *p* < 0.05 were considered statistically significant.

## Results

### NG-R1 Alleviates the Symptoms of Articular Inflammation in TNF-Tg Mice

**FIGURE 1:**
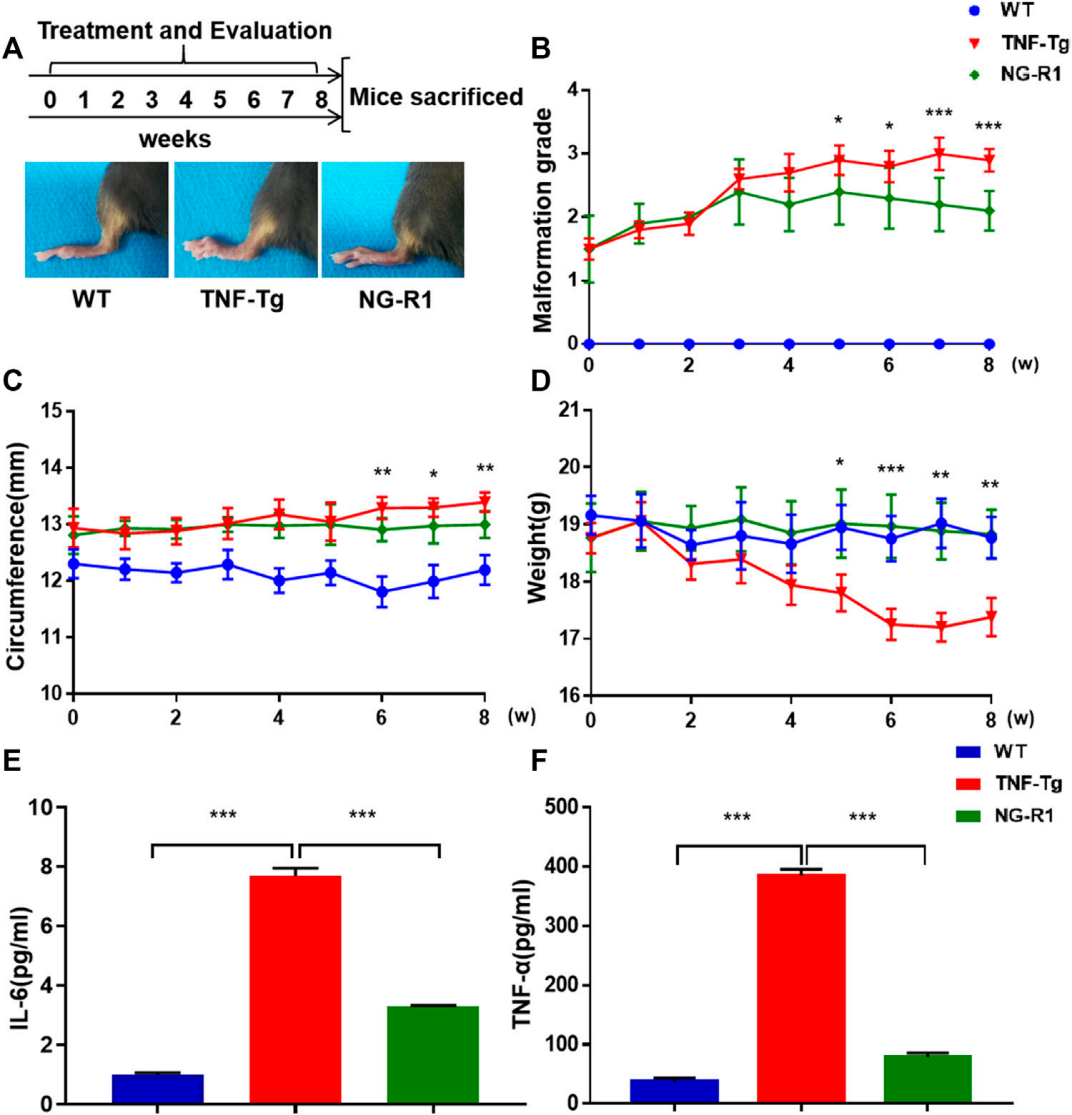
TNF-Tg mice exhibited ameliorated inflammation after 8 weeks of NG-R1 treatment. **(A)** Schematic flow of mice treatment strategy and experimental procedure. The representative pictures of the paws of mice after treatment. **(B–D)** The arthritis malformation grades,circumference and weight in TNF-Tg mice were evaluated weekly. **(E,F)** ELISA Kit was performed on serum for IL-6 and TNF-α. Data represent mean ± SEM, (*n* = 10 per group). *p < 0.05, **p < 0.01, ***p < 0.001, two-way ANOVA.

To establish whether NG-R1 reduced inflammation, we used TNF-Tg mice model and treated with NG-R1 for 8 weeks (Figure 1). As for arthritis symptoms assessment, we found that NG-R1 treatment group did significantly attenuate the degree of joint malformation and ankle circumference in TNF-Tg mice compared to the model group (Figures 1B,C). Body weight is an important index to reflect body development, and showed that NG-R1 treatment group did effectively improved the trend of weight loss in TNF-Tg mice (Figure 1D). To further confirm the anti-inflammatory effect of NG-R1 in TNF-Tg mice, we tested the expression of serum inflammatory factors in mice by ELISA Kit and found that NG-R1 could effectively inhibit the expression of serum inflammatory factors IL-6 and TNF-α (Figures 1E,F). These results indicated that NG-R1 alleviates the symptoms of articular inflammation in TNF-Tg mice.

### NG-R1 Attenuated the Synovial Inflammation, Hyperplasia, and the Cartilage and Bone Destruction of the Joint

**FIGURE 2:**
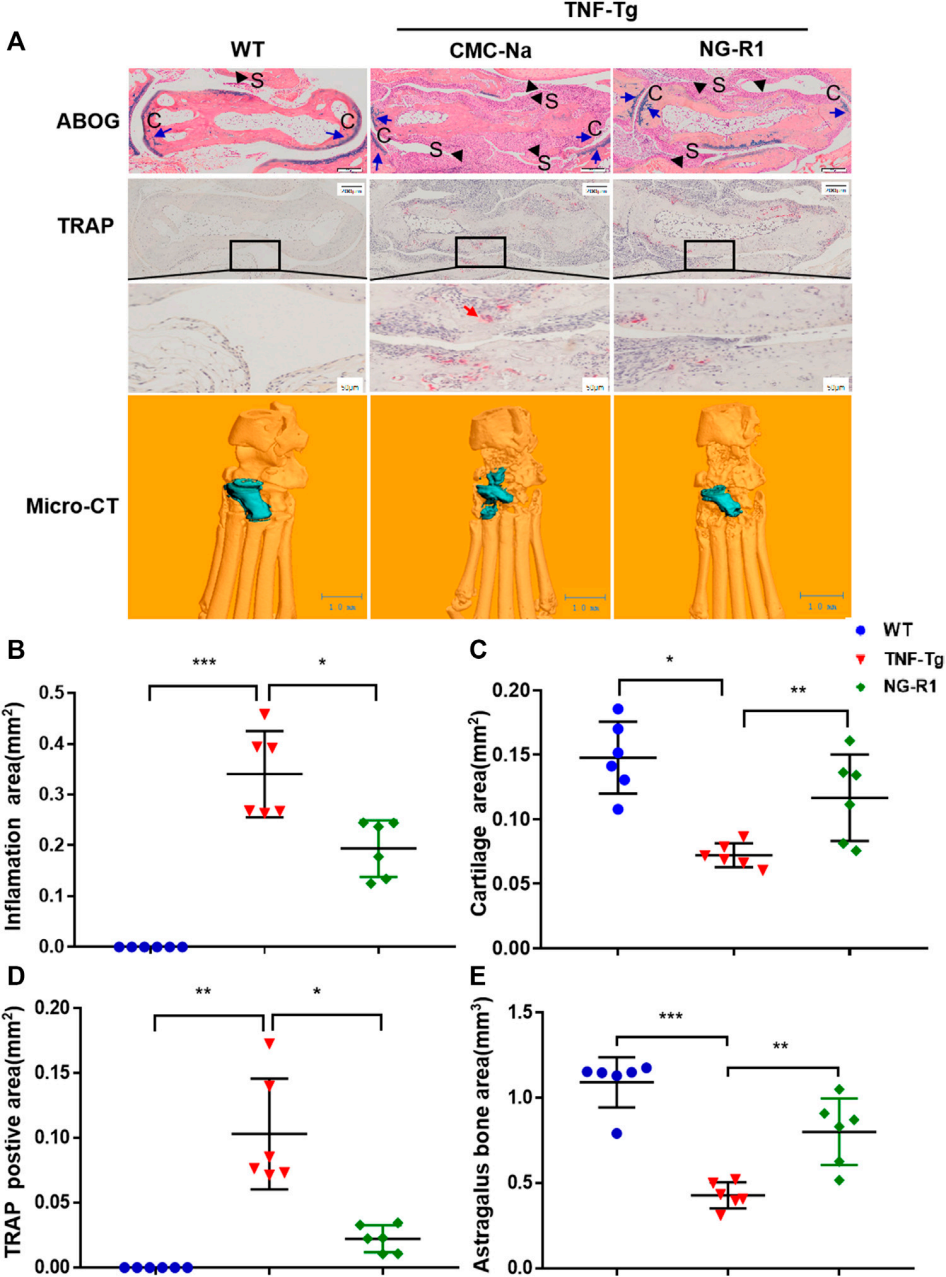
NG-R1 protects TNF-Tg mice from synovial inflammation and inflammatory bone loss. **(A)** Representative images stained for ABOG and TRAP at ankle joint sections. Black arrows indicate synovial tissue (S). Blue arrows indicate cartilage (C). Red arrows indicate the TRAP-positive mature osteoclasts. Longitudinal sections of 3-dimensional reconstructed ankle joints by micro-CT. ABOG staining: Magnification, ×80; scale bars per column, 200 μm. TRAP staining: Magnification, ×80; scale bars per column, 200 μm. Locally large graph: Magnification, ×200; scale bars per column, 50 μm. **(B–E)** Quantification of inflamation area, cartilage area, trap postive area, and astragalus bone area. Data represent mean ± SEM (*n* = 6 per group). *p < 0.05, **p < 0.01, ***p < 0.001, one-way ANOVA.

A histopathological evaluation of the ankle joints was performed to examine the degrees of inflammatory damage. Histological examination and histomorphometric analysis showed that ankle joints from the model group had severe synovial inflammatory hyperplasia, cartilage loss, bone destruction, and greater osteoclasts numbers around the astragalus. ABOG staining analysis showed that NG-R1 treatment group had conspicuous reduced synovial inflammation, bone erosion, and cartilage destruction (Figures 2A–C,E). TRAP staining found that the NG-R1 treatment group had fewer osteoclasts around the astragalus in the ankle joints than the model group (Figures 2A,D). Three-dimensional micro-CT results indicated that the ankle joints in the model group was obviously eroded, while the NG-R1 treatment group remained intact (Figure 2A). These results indicated that NG-R1 effectively attenuated the inflammatory symptoms of ankle joints and inhibited the development of rheumatoid arthritis in TNF-Tg Mice.

### NG-R1 Promotes Lymphatic Drainage Function in TNF-Tg Mice

**FIGURE 3:**
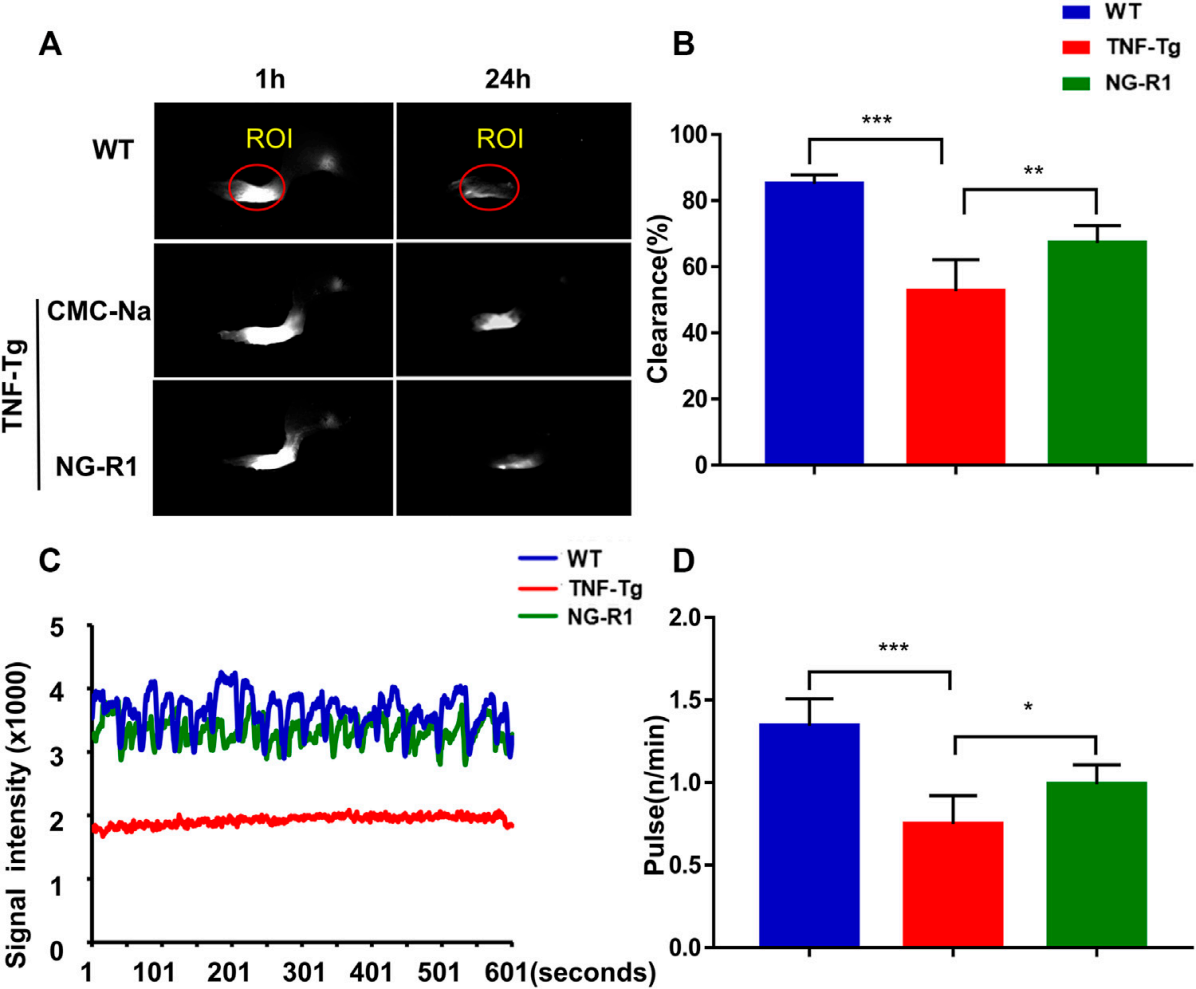
NG-R1 improved the lymphatic drainage function by increasing the pulse and clearance rate of TNF-Tg mice lymphatic vessels. Lymphatic vessels of the left footpad were examined using near-infrared ICG imaging immediately and at 1 and 24 h post-ICG administration. The ICG fluorescence signal intensity at the footpads (outlined by the red circles) was recorded and used the software ImageJ analyzes the marked area and obtains within 600 s fluctuates. **(A,B)** A representative ICG signal of footpads and the percentage of ICG clearance. **(C,D)** Detection and analysis of lower limb lymphatic contraction pulse in TNF-Tg mice. Data represent mean ± SEM (*n* = 6 per group). *p < 0.05, **p < 0.01, ***p < 0.001, one-way ANOVA.

Our previous studies demonstrated that sufficient lymph drainage helps to improve joint inflammation (Liang et al., 2015). We next sought to investigate if NG-R1 attenuated inflammation in TNF-Tg mice was somehow due to promoted lymphatic drainage function. The lymphatic vessel function was related to the contraction frequency of lymphatic vessels and lymphatic pulse frequency. At 24 h after mice were injected with ICG in the left footpad, we found that the fluorescence intensities of TNF-Tg mice were higher, and this prompted us that TNF-Tg mice display impaired lymphatic function. However, after NG-R1 treatment the mice showed more rapid clearance of ICG, and this results indicates an enhanced lymphatic clearance function (Figures 3A,B). Lymphatic pulse frequency is proven to be another important indicator of lymphatic drainage function. The lymphatic pulse frequency of TNF-Tg mice was about 0.7 beats per minute, and then after NG-R1 treatment, the frequency was able to reach 1.0 beats per minute (Figures 3C,D). These data suggested that NG-R1 promotes lymphatic drainage function in TNF-Tg mice.

### NG-R1 Improves Lymphatic Vessels Structure Recovery

**FIGURE 4:**
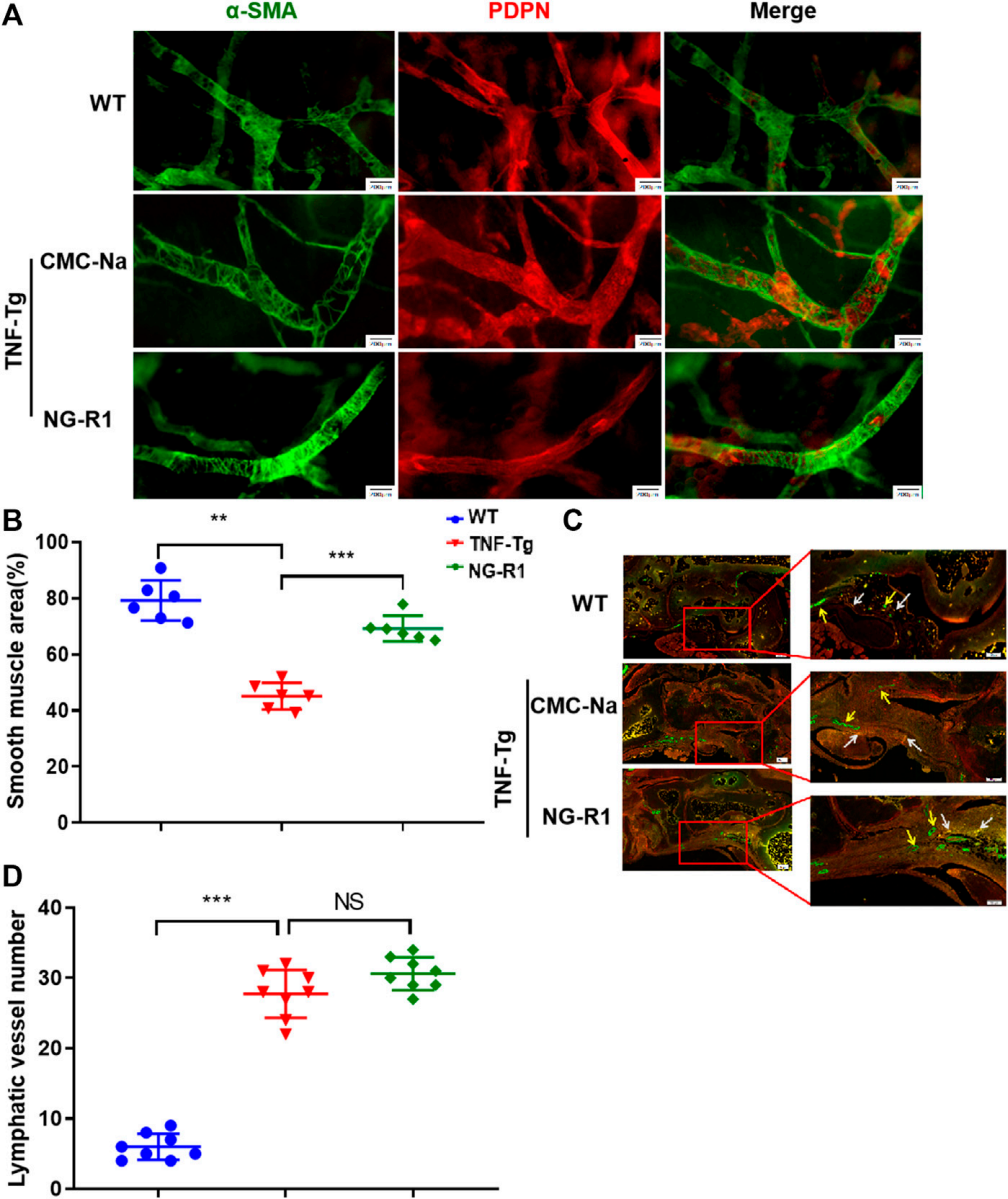
NG-R1 improves lymphatic vessels structure recovery. **(A)** Whole Mount Immunofluorescence Staining of Lymphatic capillaries. α-SMA (green)and PDPN (red). Bar, 200 μm. **(B)** Proportion of lymphatic smooth muscle region were counted (*n* = 6 per group). **(C)** Immunofluorescence Staining showed that yellow arrow was indicated blood vessels (Podoplanin−/α-SMA+), white arrow indicates collecting lymphatic vessels (Podoplanin+/SMA+). **(D)** The number of lymphatic vessels was statistically analyzed (*n* = 8 per group). Immunofluorescence staining: Magnification, ×80; scale bars per column, 200 μm. Locally largegraph: Magnification, ×80; 100; scale bars per column, 100 μm. Data represents mean ± SEM, NS > 0.05, *p < 0.05, **p < 0.01, ***p < 0.001, One-way ANOVA.

To prove whether the mechanism of NG-R1 improving lymphatic function is related to promoting lymphatic structural recovery and increasing the number of lymphatic vessels, we performed Whole Mount staining and immunofluorescence staining. Whole Mount staining revealed that the structure of the lymphatic vessel was significant destruction and the area covered by lymphatic smooth muscle was reduced in the model group (Figure 4A). The area covered by lymphatic smooth muscle was increased in the NG-R1 treatment group compared with the TNF-Tg mice group (Figure 4B). In addition, we conducted podoplanin/α-SMA immunofluorescence staining to examine the numbers of the lymphatic vessel. Immunofluorescence staining is visible in yellow indicating the collecting lymphatic vessels (Figure 4C). We performed statistical experiments on the number of collecting lymphatic vessels. However, NG-R1 did not affect the lymphatic vessel numbers in TNF-Tg mice (Figure 4D). These data indicate that NG-R1 improves lymphatic vessel structure recovery but not increase the number of lymphatic vessels.

**FIGURE 5:**
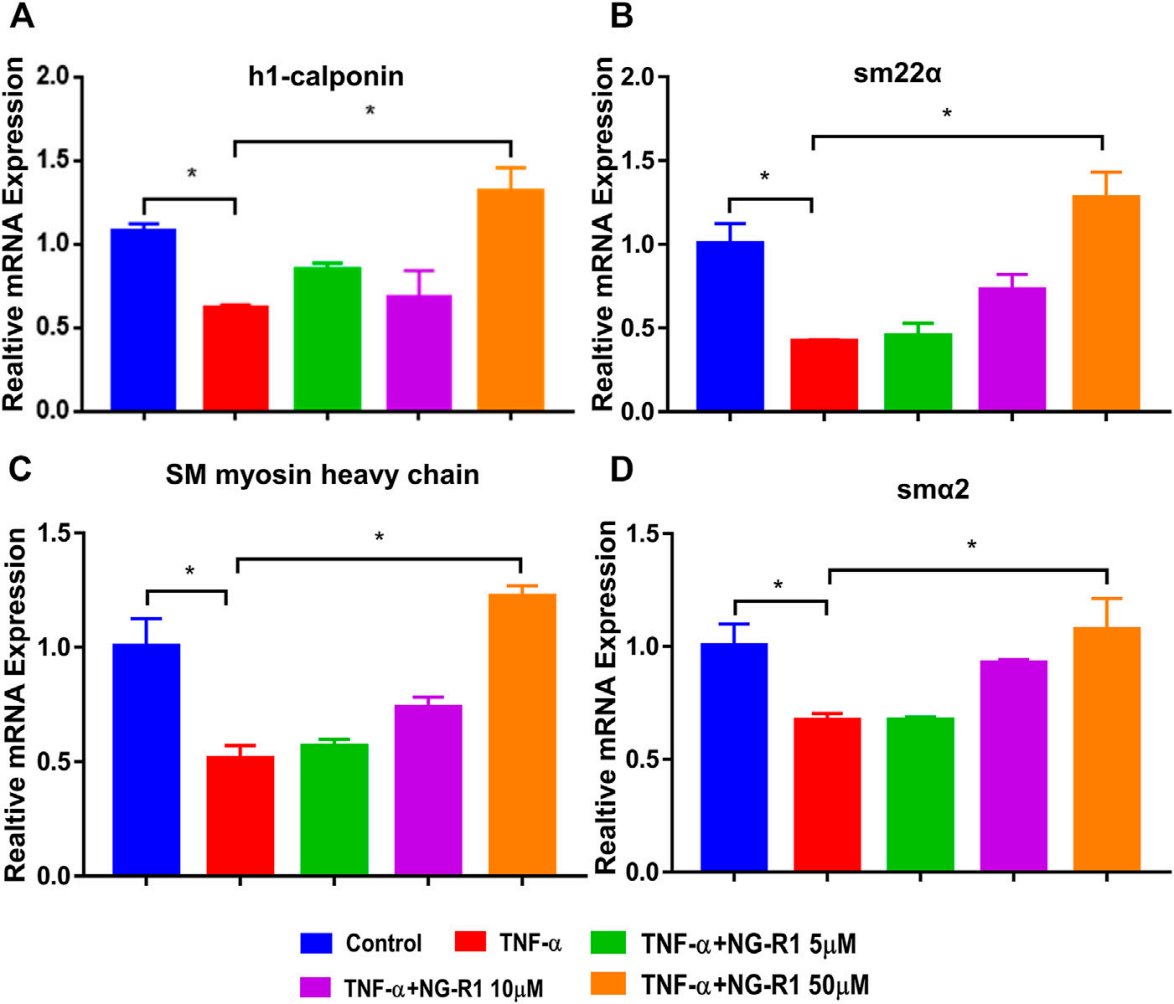
NG-R1 improved the function of lymphatic smooth muscle cells (LSMCs) in the inflammatory state induced by TNF-α. **(A–D)** The coculture model of LECs and LSMCs was used to detect the effect of NG-R1 (5, 10, and 50 μM) on the expression level of functional muscle genes h1-calponin, smα22, SM myosin heavy chain, smα2 mRNA of LSMCs in inflammatory environment induced by TNF-α (20 ng/ml). Data represents mean ± SEM. The experiment was repeated at least three times. *p < 0.05, One-way ANOVA.

To further verify the effect of NG-R1 on lymphatic smooth muscle cell function, we simulated the lymphatic structure and established a coculture system. LECs with or without NG-R1 pretreatment were stimulated with TNF-α and then co-cultured with LSMCs via transwell for 24 h. The results demonstrated that paracrine factors from the TNF-α stimulated LECs reduced the expression levels of muscle-related genes, including h1-calponin, smα22, smooth muscle myosin heavy chain, and smα2 in the LSMCs, which is prevented by NG-R1 (Figures 5A–D). NG-R1 increased the expression of the lymphatic smooth muscle cell function marker gene and promoted the recovery of the lymphatic vessel structure.

### NG-R1 Reduces the Production of Pro-Inflammatory Cytokines by TNF-α Treated LECs

**FIGURE 6:**
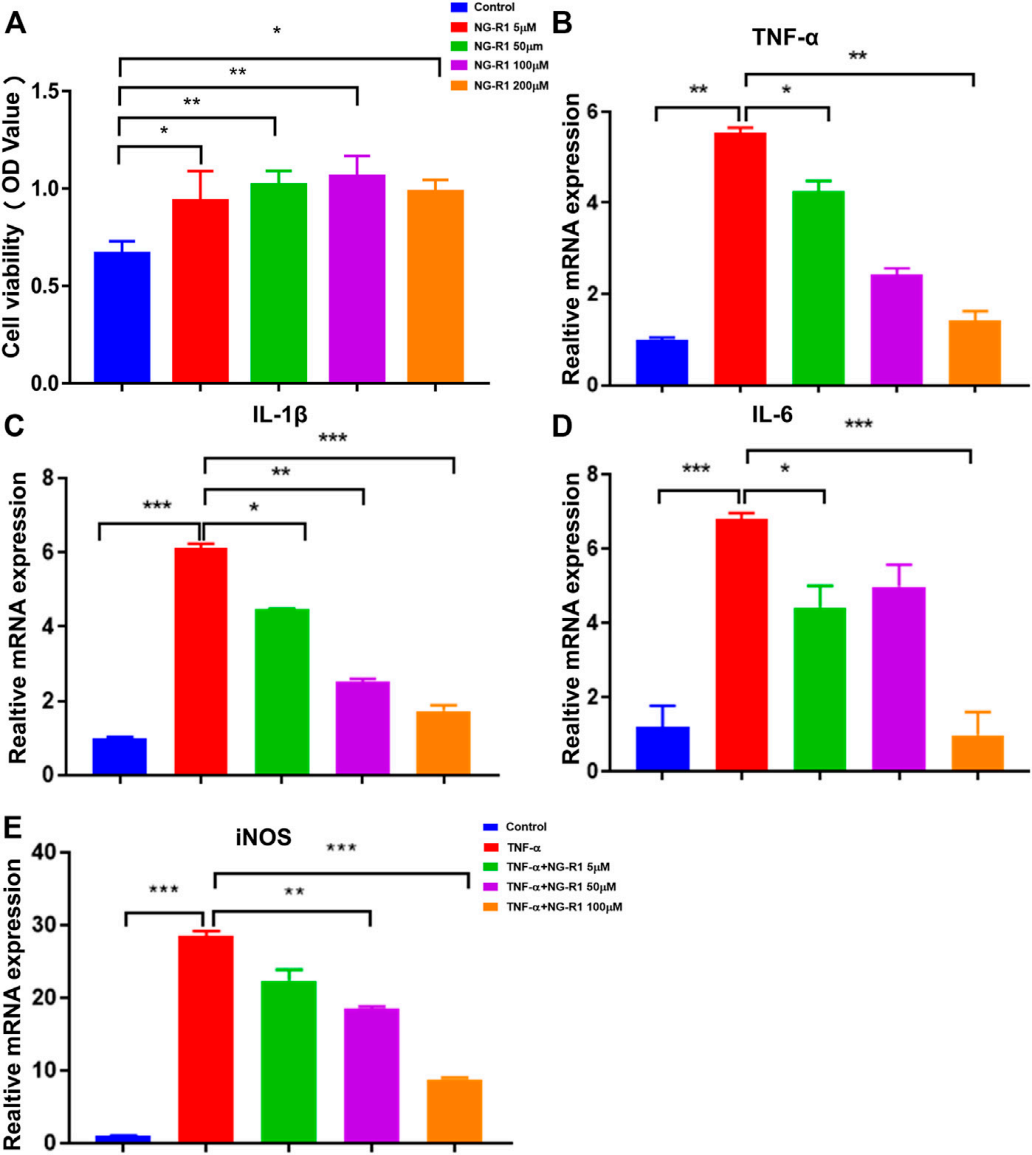
NG-R1 ameliorated the inflammatory cytokines production of LECs stimulated by TNF-α. **(A)** Cytotoxicity assay used Cell Counting Kit-8 to determine the effective concentration of NG-R1. LECs was treated with different concentrations of NG-R1 (5, 50, 100, and 200 μM) for 24 h. **(B–E)** LECs were also pretreated for 2 h with NG-R1 (5, 50, and 100 μM) prior to stimulation with TNF-α (20 ng/ml) for another 12 h. The mRNA levels of TNF-α,IL-1β, IL-6, and iNOS were determined using qPCR. Data represents mean ± SEM. The experiment was repeated at least three times. *p < 0.05, **p < 0.01, ***p < 0.001, One-way ANOVA.

We investigated the CCK-8 kit to assess the potential cytotoxicity of NG-R1, and we found that NG-R1 did not affect cell viability on LECs after incubation 24 h even at a concentration of 100 µM (Figure 6A). Therefore, we decided to set the highest concentration of NG-R1 at 100 µM for the following experiments. LECs were pretreated with NG-R1 (5, 10, and 50 µM) for 2 h, then stimulated with TNF-α (20 ng/ml) for another 12 h. It has been shown that TNF-α significantly increased crucial pro-inflammatory cytokines TNF-α, IL-1β, IL-6, and iNOS mRNA expression levels. We found that NG-R1 dose-dependently decreased the mRNA expression of TNF-α, IL-1β, IL-6, and iNOS induced by TNF-α activated LECs (Figures 6B–E).

### NG-R1 Suppressed the Phosphorylation of p65 and p65 Nuclear Accumulation in TNF-α-Induced LECs

**FIGURE 7:**
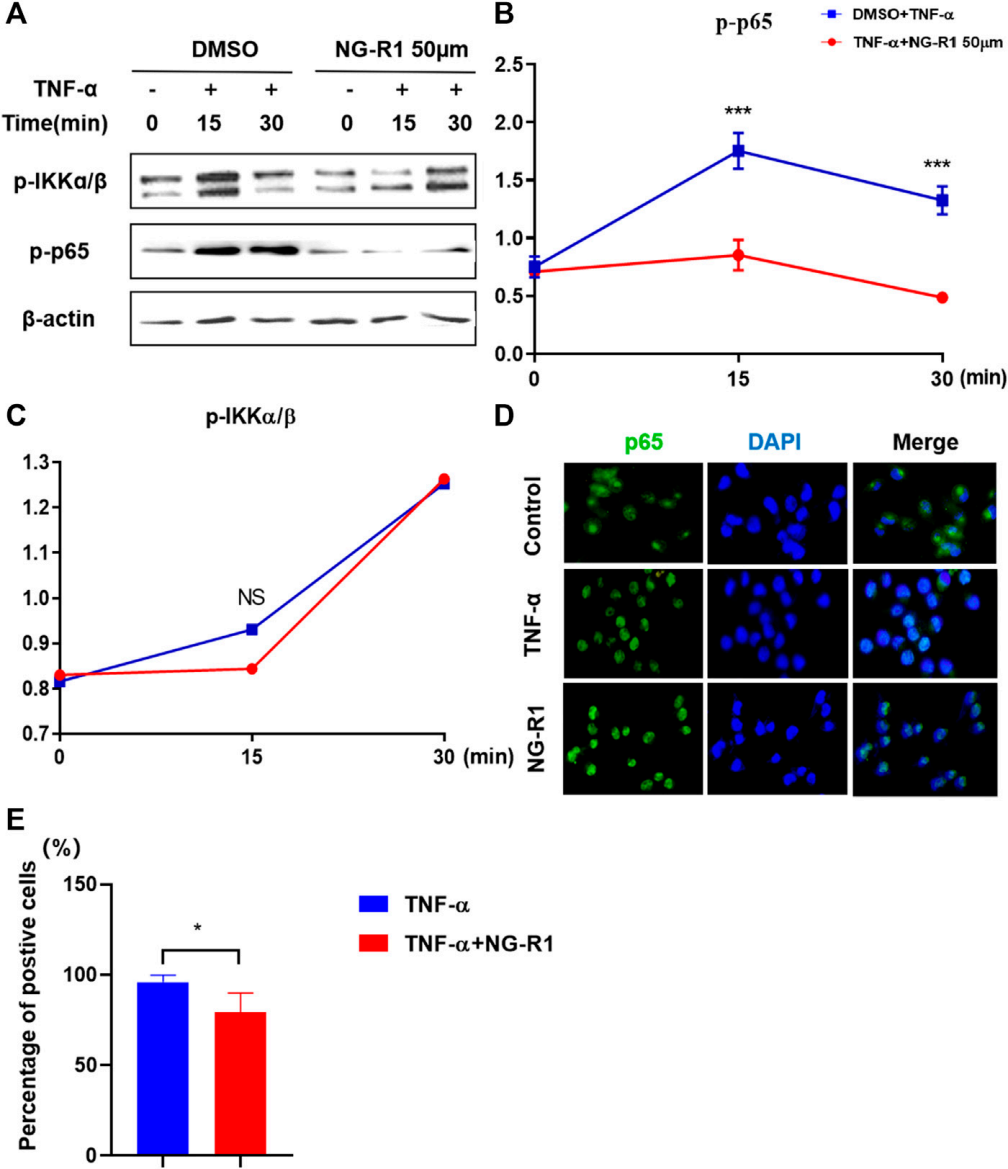
NG-R1 inhibited NF-κB activation by impairing p65 phosphorylation. **(A–C)** Immunoblots for phos-phorylation of IKKα/β, p65, protein level in LECs stimulated with TNF-α (20 ng/ml) for 0, 15, and 30 min in the presence of NG-R1 (50 µM). The experiment was repeated at least three times. **(D)** The cells were stained with anti-p65 (green) and DAPI (blue) for 30 min. **(E)** LECs were stimulated with TNF-α for 30 min in the presence or absence of NG-R1 pretreatment for 2 h. Data represent mean ± SEM. The experiment was repeated at least three times. *p < 0.05, one-way ANOVA.

It has been demonstrated that the NF-κB signaling pathway is a crucial inflammatory pathway involved in the development of rheumatoid arthritis. Thus, we examined whether NG-R1 regulates the activation of NF-κB signaling in LECs cells stimulated with TNF-α. We found that the phosphorylation of IKKα/β and p65 was markedly increased after being stimulated with TNF-α (20 ng/ml) for 15 min and 30 min. The phosphorylation of p65 was significantly inhibited in TNF-α-induced cells by treatment of NG-R1 (50 µM). However, it did not affect the phosphorylation of IKKα/β (Figures 7A–C). In addition, an immunofluorescence assay revealed that NG-R1 significantly reduced the level of p65 in the nucleus of LECs induced TNF-α-stimulated (Figures 7D–E). Thus, NG-R1 inhibited TNF-α-induced activation of the NF-κB signaling pathway.

## Discussion

NG-R1 exhibits a pharmacological activity against excessive inflammation, but its mechanism of action has not been studied in RA. Here, our study is the first time to investigate the anti-inflammatory effect of NG-R1 in RA as well as elucidate its possible mechanism by which NG-R1 alleviates RA symptoms. Our current study demonstrated NG-R1 as a potential effective therapeutic medicine for the treatment of RA. We found that NG-R1 effectively improved inflammatory symptoms in the ankle joint of TNF-Tg mice by promoting the lymphatic drainage function while suppressing NF-κB signaling pathway-mediated production of proinflammatory effectors in LECs.

Notoginsenoside (NG-R1) is a novel triterpene saponin compound, one of the main bioactive ingredients from Panax notoginseng (PN) root, which is commonly used to treat cardiovascular diseases (Zhu et al., 2020), neurodegenerative disease (Liu et al., 2020), diabetic nephropathy (Zhang et al., 2019), and cancer (Li et al.,2020). NG-R1 improved the imbalance between iNOS and eNOS and blockaded activation of NF-κB and the subsequent myocardial inflammatory and apoptotic responses in endotoxemic mice (Sun et al., 2013). NG-R1 relived LPS-elicited inflammatory damages via blocking NF-κB in a miR-301a-silenced manner (Dong et al., 2020). NG-R1 protected neuron cells from inflammatory damage by decreasing pro-inflammatory cytok IL-2, IL-6, and TNF-α expression, and inactivating the JNK pathway (Sun et al., 2019). Inflammation plays a key role in its pathogenesis, and they can aggravate persistent pain, structural change within the joint, bone destruction, and disability. TNF-Tg mice as a mature model of RA with TNF-α overexpression, which spontaneously develop mild ankle joint inflammation and bone erosion (Wang et al., 2020). Therefore, we chose 3-month-old TNF-Tg mice and WT littermates, and our current study proved that NG-R1 significantly attenuated the degree of joint malformation and ankle swelling in TNF-Tg mice, and improved the gradual emaciation of mice. The pathological changes of rheumatoid arthritis usually include synovial hyperplasia (Nygaard and Firestein et al., 2020), pannus formation (Huh et al., 2015), degeneration of cartilage, and bone destruction (Hardy et al., 2018). histopathological evaluation of the ankle joints and three-dimensional micro-CT further confirmed that synovial hyperplasia, cartilage loss, and bone destruction were effectively relieved after the treatment of NG-R1. Evidence suggests that cytokines such as IL-1, IL-6, and TNF-α contribute to the induction and maintenance of inflammation during the pathological process of RA (Noack and Miossec et al., 2017). Meanwhile, our study confirmed that NG-R1 inhibited the expression of inflammatory factors IL-6 and TNF-α in serum and reduced the inflammatory response *in vivo*.

The lymphatic system plays an integral role in the development and progression of a range of disease conditions (Abdallah et al., 2020), which maintains extracellular fluid homeostasis as favorable, promotes metabolism, regulates immunity, and this also proves that it is possible to study the pathology and mechanism of the disease from the perspective of the lymphatic system (Breslin et al., 2018). We have shown previously that TNF-Tg mice with severe arthritis typically have reduced or loss of lymphatic vessel contractions and decreased lymphatic flow from inflamed joints, but the mechanisms involved are unknown. Our previous studies showed that sufficient lymph drainage helps to improve joint inflammation, and drugs activating lymphatic function can attenuate synovial inflammation (Guo et al., 2009). In this study, we showed NG-R1 promoted lymphatic drainage function in TNF-Tg mice by accelerating lymphatic pulse frequency and lymphatic clearance. Next, we investigated the effect of NG-R1 on the structure and number of lymphatic vessels. Immunofluorescence staining results confirmed that NG-R1 did not affect the lymphatic vessel numbers in TNF-Tg mice, but it improved the destruction of lymphatic structures in the inflammatory state and increased the area covered by lymphatic smooth muscle.

Our previous studies have confirmed the importance of lymphatic system in arthritis inflammation. In this study, as a common inflammatory inducer, TNF-α was applied to evoke extreme inflammation in LECs. TNF promoted the production of nitric oxide (NO) by LECs and caused LSMCs apoptosis, reduced expression of muscle functional genes, and LSMCs contribute to the dysfunction of synovial lymphatic vessels in inflammatory arthritis. Thus, we conducted the coculture system of LECs and LSMCs.The results demonstrated that NG-R1 increased the expression of lymphatic smooth muscle cell function marker geneh1-calponin, smα22, SM myosin heavy chain, and smα2 and promoted the contractile function of LSMCs and accelerated lymphatic drainage. NF-κB signaling pathway is one of the important transcriptional pathways in RA, which mainly regulates the level of pro-inflammatory mediators and induces the expression of IL-1, IL-6, and TNF-α and promotes the phosphorylation of p65 and IKKα/β in LECs. Our results show that NG-R1 dose-dependently inhibits TNF-α induced inflammatory cytokines TNF-α, IL-1β, IL-6, and iNOS production in LECs. NG-R1 repressed the phosphorylation of p65, and p65 nuclear accumulation relieved TNF-α-elicited inflammatory damages. However, NG-R1 without any effect on the phosphorylation of IKKα/β under the stimulation of TNF-α. Furthermore, inhibiting NF-κB signaling pathway in TNF-TG mice also inhibited the expression of serum inflammatory factors and improved synovial inflammation, cartilage erosion, and bone destruction of the ankle joint. These have led us to speculate that NG-R1 may play an anti-inflammatory role in RA. In future work, we also plan to continue to conduct experiments to explore NF-κB signal pathways by adding inhibitors or activators.

## Conclusion

In summary, using TNF-Tg mice as a model of chronic inflammatory arthritis, we reported for the first time that NG-R1 promoted lymphatic drainage function to ameliorate rheumatoid arthritis by suppressing the NF-κB signaling pathway. Thus, inhibiting inflammatory cytokines production and improvement of the lymphatic vessel draining function together represent a potential therapeutic strategy for RA. Meanwhile, it also represents that NG-R1 is a new potential treatment for rheumatoid arthritis.

## Data Availability

The original contributions presented in the study are included in the article/Supplementary Material, and further inquiries can be directed to the corresponding authors.
