# Supplementary figures and images for: Notoginsenoside R1 (NG-R1) Promoted Lymphatic Drainage Function to Ameliorating Rheumatoid Arthritis in TNF-Tg Mice by Suppressing NF-κB Signaling Pathway
Source: Front Pharmacol. 2022 Feb 24;12:730579. doi: 10.3389/fphar.2021.730579 (PMC8909130; doi:10.3389/fphar.2021.730579)

## Slide 1
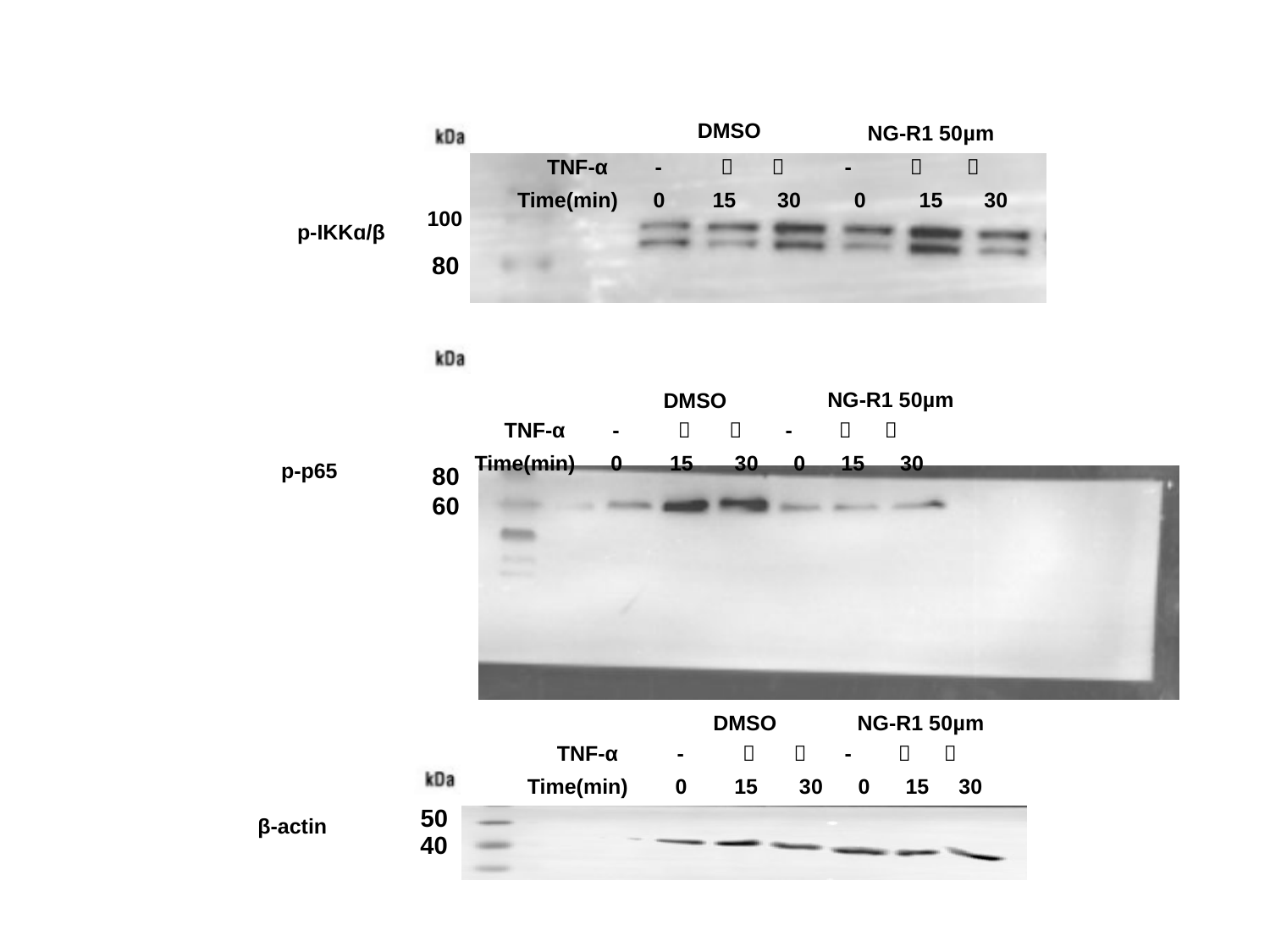

DMSO
NG-R1 50µm
p-IKKɑ/β
100
80
 TNF-α - ＋ ＋ - ＋ ＋
Time(min) 0 15 30 0 15 30
NG-R1 50µm
 DMSO
 TNF-α - ＋ ＋ - ＋ ＋
Time(min) 0 15 30 0 15 30
p-p65
80
60
 DMSO
NG-R1 50µm
 TNF-α - ＋ ＋ - ＋ ＋
Time(min) 0 15 30 0 15 30
50
β-actin
40

Supplement: Supplementary file 1 [file Presentation1.PPTX]

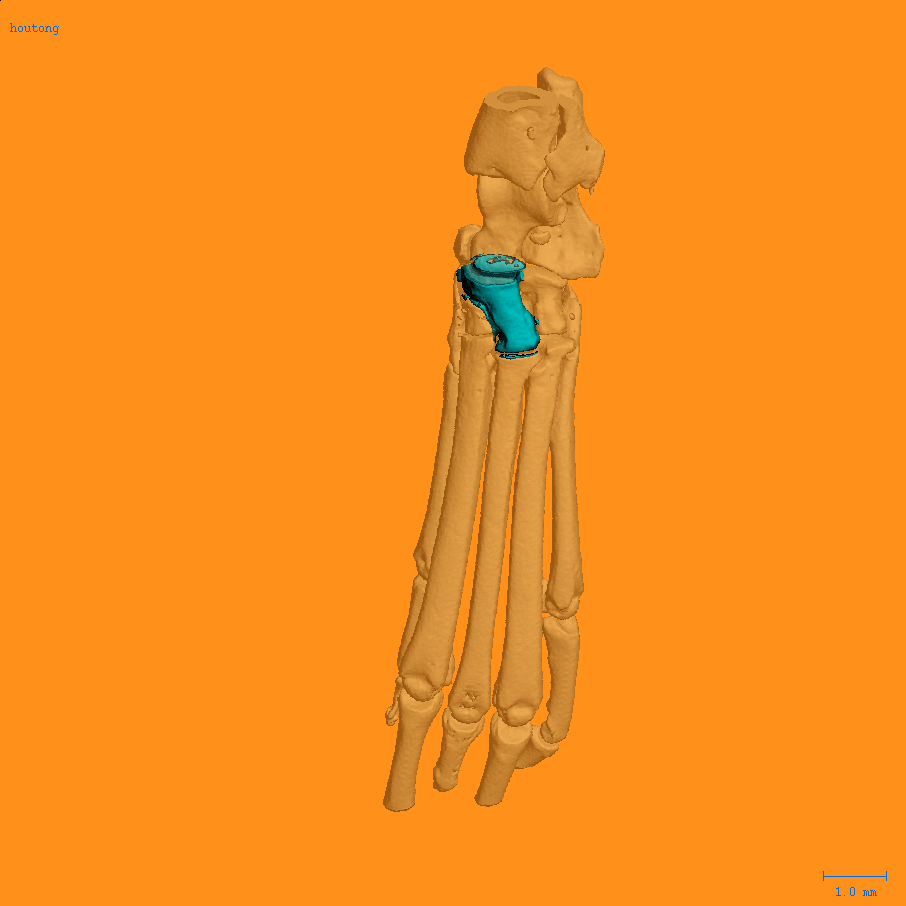

Supplement: Supplementary file 2 [file Image3.TIF]

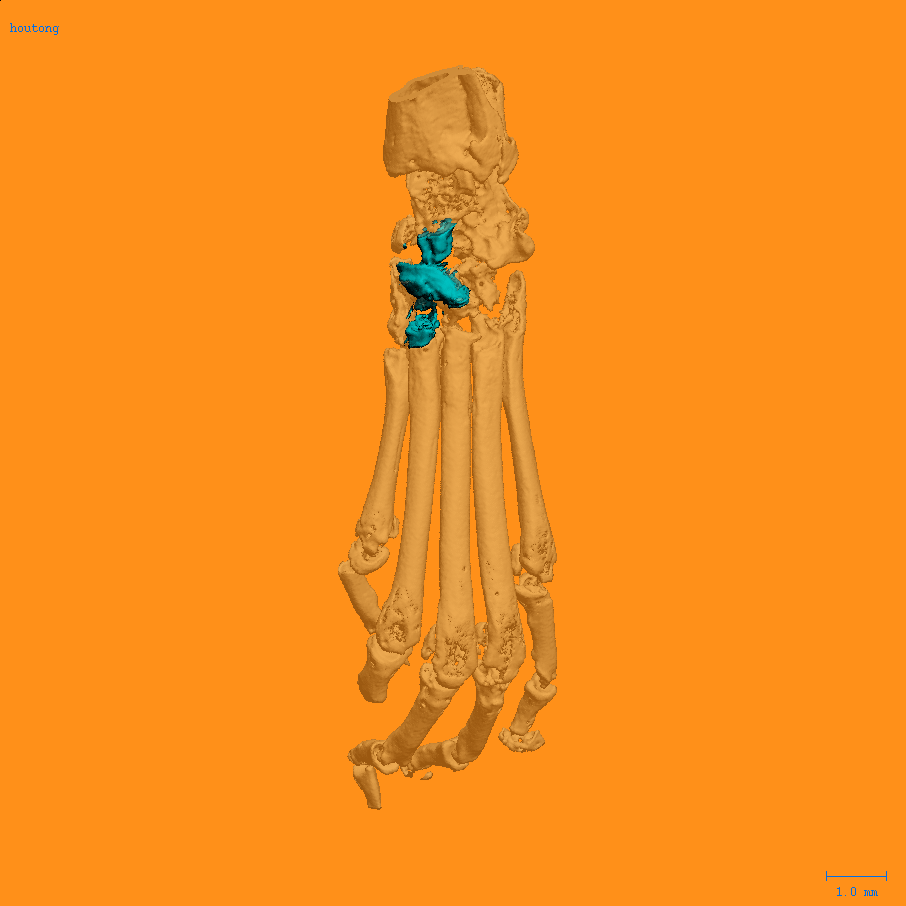

Supplement: Supplementary file 3 [file Image2.TIF]

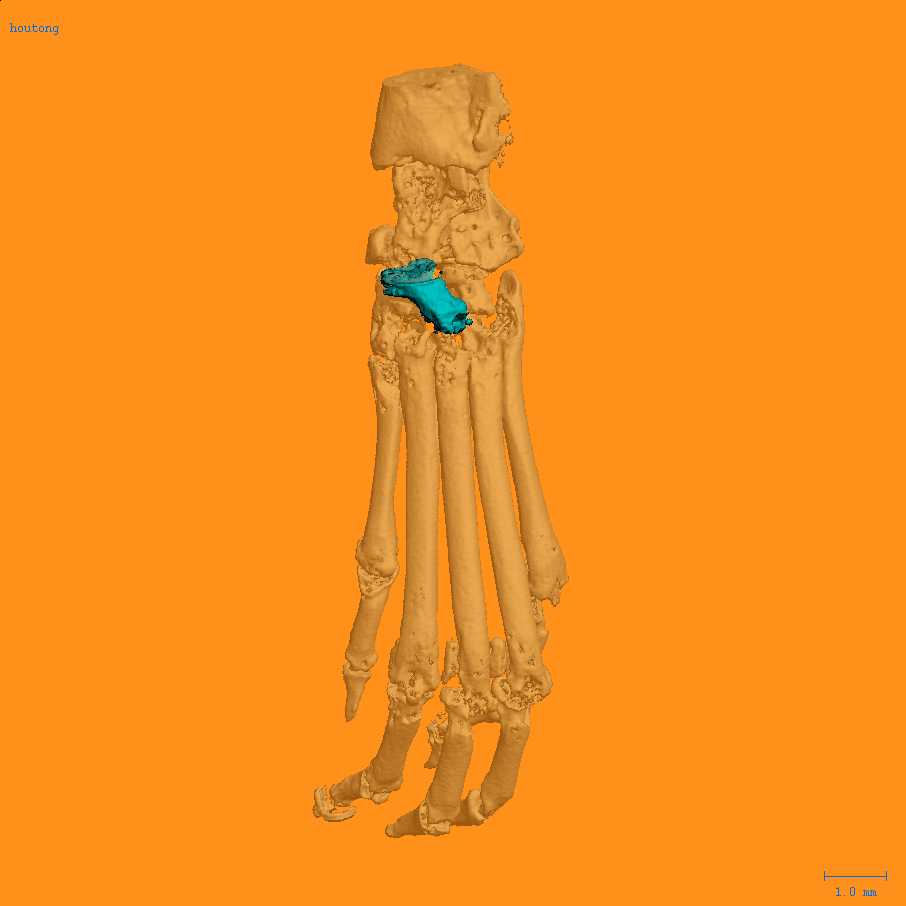

Supplement: Supplementary file 4 [file Image1.TIF]
